# Pachychoroid as a Risk Factor for Exudative Retinal Detachment After Panretinal Photocoagulation: A Report of Two Cases

**DOI:** 10.7759/cureus.73228

**Published:** 2024-11-07

**Authors:** Rituraj P Videkar, Hassan Salim Al Hasid, Mohammad Fazal Kamal, Gangaprasad Amula, Mandeep Lamba

**Affiliations:** 1 Ophthalmology, Fakeeh University Hospital, Dubai, ARE; 2 Ophthalmology, Dubai Hospital, Dubai, ARE; 3 Ophthalmology, Al-Ahli Hospital, Doha, QAT; 4 Ophthalmology, Prime Hospital, Dubai, ARE

**Keywords:** exudative retinal detachment, laser complication, pachychoroid, panretinal photocoagulation laser, proliferative diabetic retinopathy (pdr)

## Abstract

In this case series of two patients, we discuss pachychoroid as a risk factor for predicting exudative retinal detachment (RD) after panretinal photocoagulation (PRP). The first patient was a 55-year-old diabetic male with unstable proliferative diabetic retinopathy (PDR), serous pigment epithelial detachment (PED), and pachychoroid confirmed via fluorescein angiography (FA) and optical coherence tomography (OCT), who underwent PRP. Post-PRP, the patient complained of visual loss in both eyes. Subsequent FA and OCT confirmed the presence of exudative RD, which resolved after a course of non-steroidal anti-inflammatory eyedrops. The second patient was a 50-year-old male with PDR, serous PED, and pachychoroid confirmed via OCT, who underwent PRP. Post-PRP, he had reduced vision due to exudative RD. His vision improved upon the resolution of the exudative RD after three weeks. Pachychoroid is known to be associated with PDR. In the presence of pachychoroid, PRP-induced inflammation overwhelms the retinal pigment epithelium due to preexisting choroidal thickening, leading to exudative RD. These cases highlight how the identification of pachychoroid before laser PRP can help in predicting exudative RD as a post-procedure complication.

## Introduction

The vascular network of the choroid, the vascular layer of the eye, comprises the choriocapillaris, Sattler’s layer, and Haller’s layer. The thickness of the choroid varies from 0.25 mm around the optic nerve to almost 0.11 mm anteriorly. A subfoveal choroidal thickness of >390 µm is known as pachychoroid [1], whose disease spectrum is associated with various retinal pathologies such as central serous retinopathy, idiopathic choroidal vasculopathy, and submacular detachment with macular edema [2]. Choroidal thickness depends on many factors, such as age, hypertension, atherosclerosis, progression of diabetic retinopathy, panretinal photocoagulation (PRP), and renal status. Proliferative diabetic retinopathy (PDR) is associated with retinal ischemia. These retinal ischemic areas are treated with PRP or laser ablation of the retina. PRP induces inflammation at the site of laser ablation, which results in scarring of the retina. Thus, PRP converts retinal ischemic areas into anoxic areas, leading to the resolution of PDR. PRP is the mainstay of treatment in PDR, with exudative retinal detachment (RD) being a rare post-PRP complication (0.07% of cases); however, the aforementioned complication appears to be an isolated event with the advent of pattern scan laser (PASCAL) (1 in 1301 consecutive cases) [3]. Exudative RD after PRP is often reported in young patients with poor glycemic control [3] and usually resolves spontaneously, but there are isolated reports of poor recovery [4]. Pachychoroid is seen in Vogt-Koyanagi-Harada syndrome, which is associated with exudative RD. However, post-PRP exudative RD and its association with pachychoroid have not been reported in the literature. Herein, we report two cases of exudative RD post-PASCAL PRP in a case of PDR with pachychoroid, which, to our knowledge, is the first such report. Identification of pachychoroid prior to PRP can help in predicting and preventing post-PRP exudative RD.

## Case presentation

Case 1

**Figure 1:**
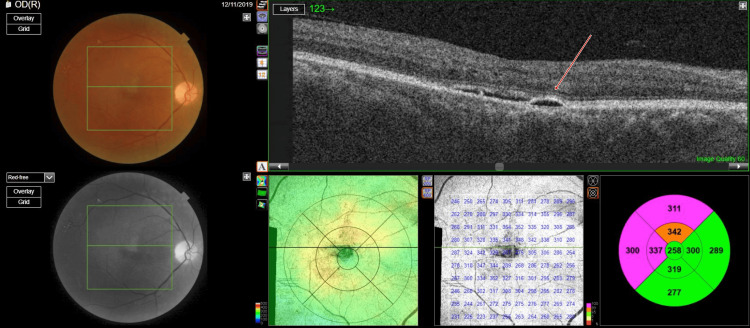
Photograph of right eye fundus and optical coherence tomography macula scan showing trace subretinal fluid with serous pigment epithelial detachment and normal foveal contour

**Figure 2:**
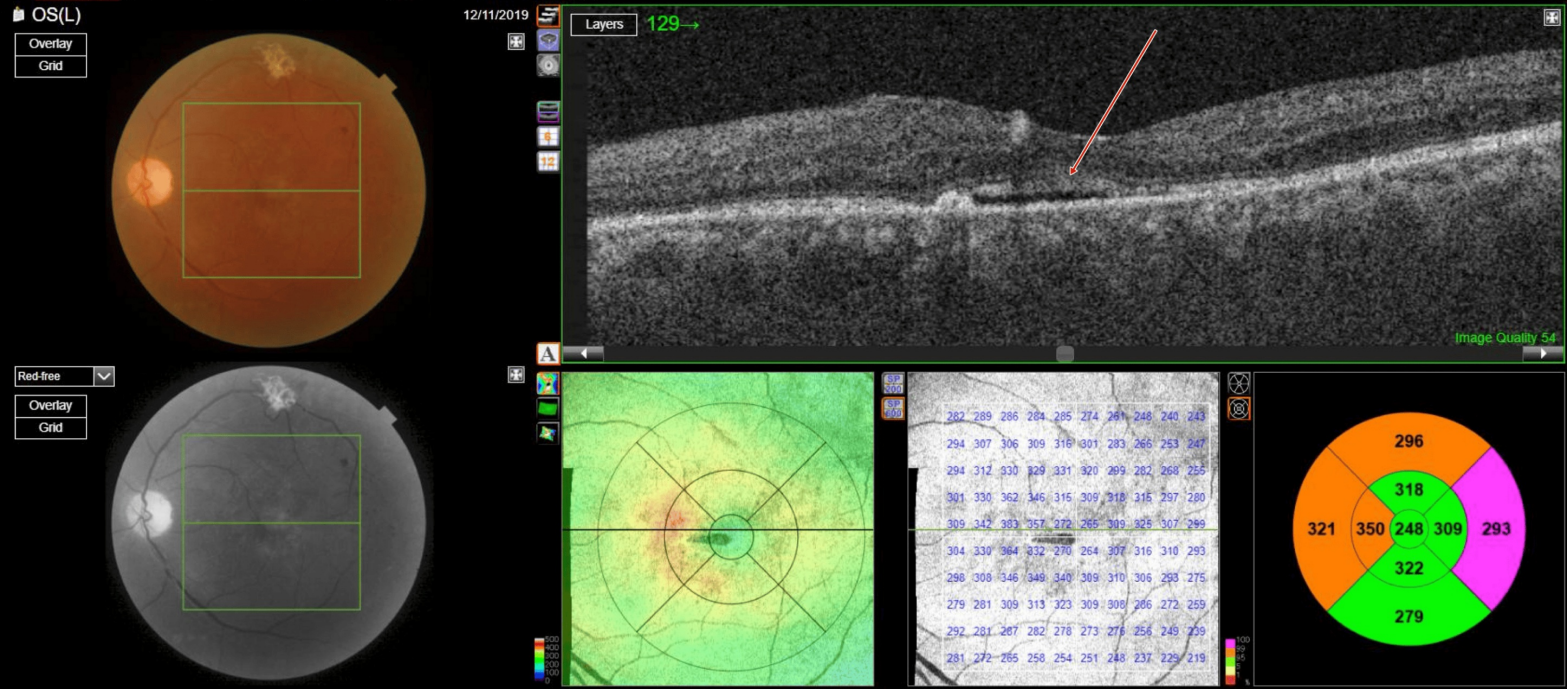
Photograph of left eye fundus and optical coherence tomography macula scan showing trace subretinal fluid with serous pigment epithelial detachment and normal foveal contour

A 55-year-old diabetic male presented to us for management of diabetic retinopathy. He gave a prior history of PRP, which was unable to stabilize his PDR due to inadequate glycemic control (HbA1C = 7.2%). He did not suffer from any other co-morbidities. His presenting visual acuity on a decimal scale was 0.9, and his intraocular pressure (IOP) was 12 mmHg in both eyes. Anterior segment examination revealed grade 2 nuclear sclerosis in both eyes, with the rest of the findings being normal. Fundus examination revealed features suggestive of lasered, unstable PDR in both eyes (OU) (Figures 1-2).

**Figure 3:**
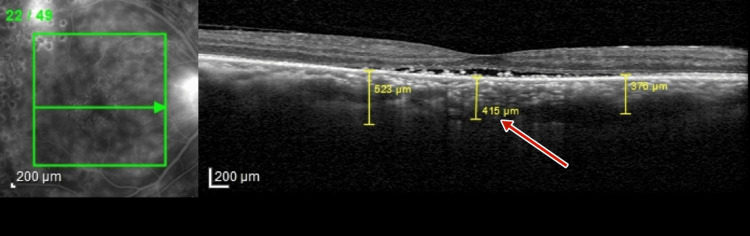
Pre-panretinal photocoagulation optical coherence tomography of right eye showing a subfoveal choroidal thickness of 415 µm suggestive of pachychoroid

**Figure 4:**
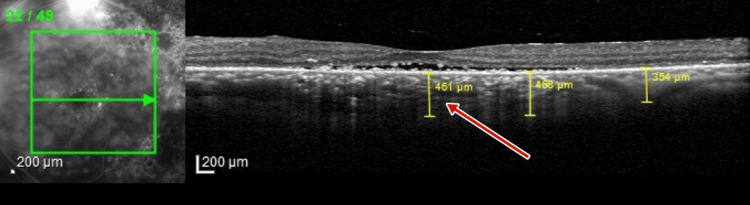
Pre-panretinal photocoagulation optical coherence tomography of left eye showing a subfoveal choroidal thickness of 451 µm, suggestive of pachychoroid

Optical coherence tomography (OCT) (TOPCON, Tokyo, Japan) examination revealed extrafoveal serous pigment epithelial detachment (PED), trace subretinal fluid in both eyes, a central retinal thickness of 258 µm in the right eye and 248 µm in the left eye, and a subfoveal choroidal thickness of 415 µm in the right eye and 461 µm in the left eye, suggestive of pachychoroid (Figures 3-4).

**Figure 5:**
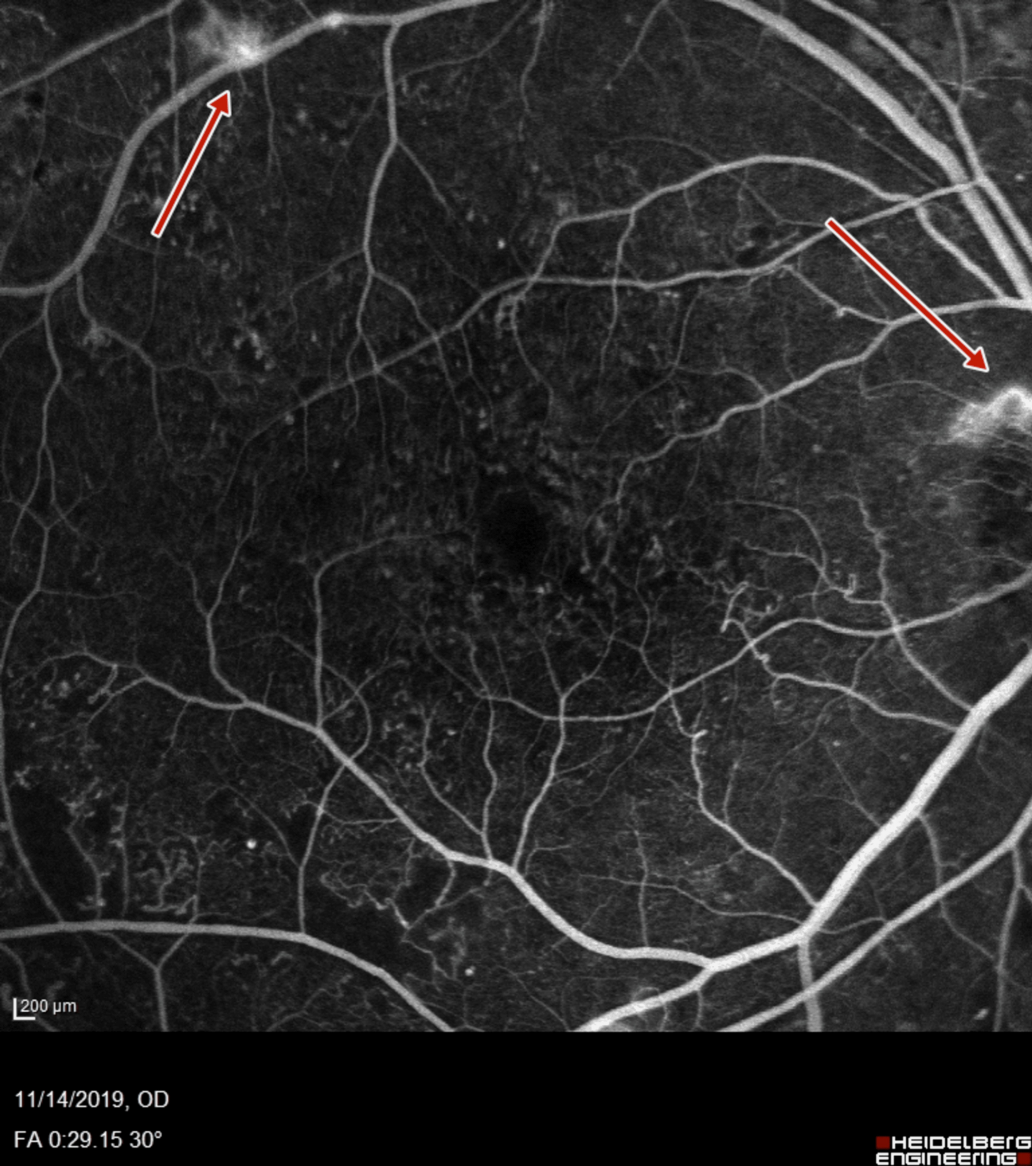
Right-eye fluorescein angiography showing neovascularization of disc and retinal neovascularization along superotemporal arcade

**Figure 6:**
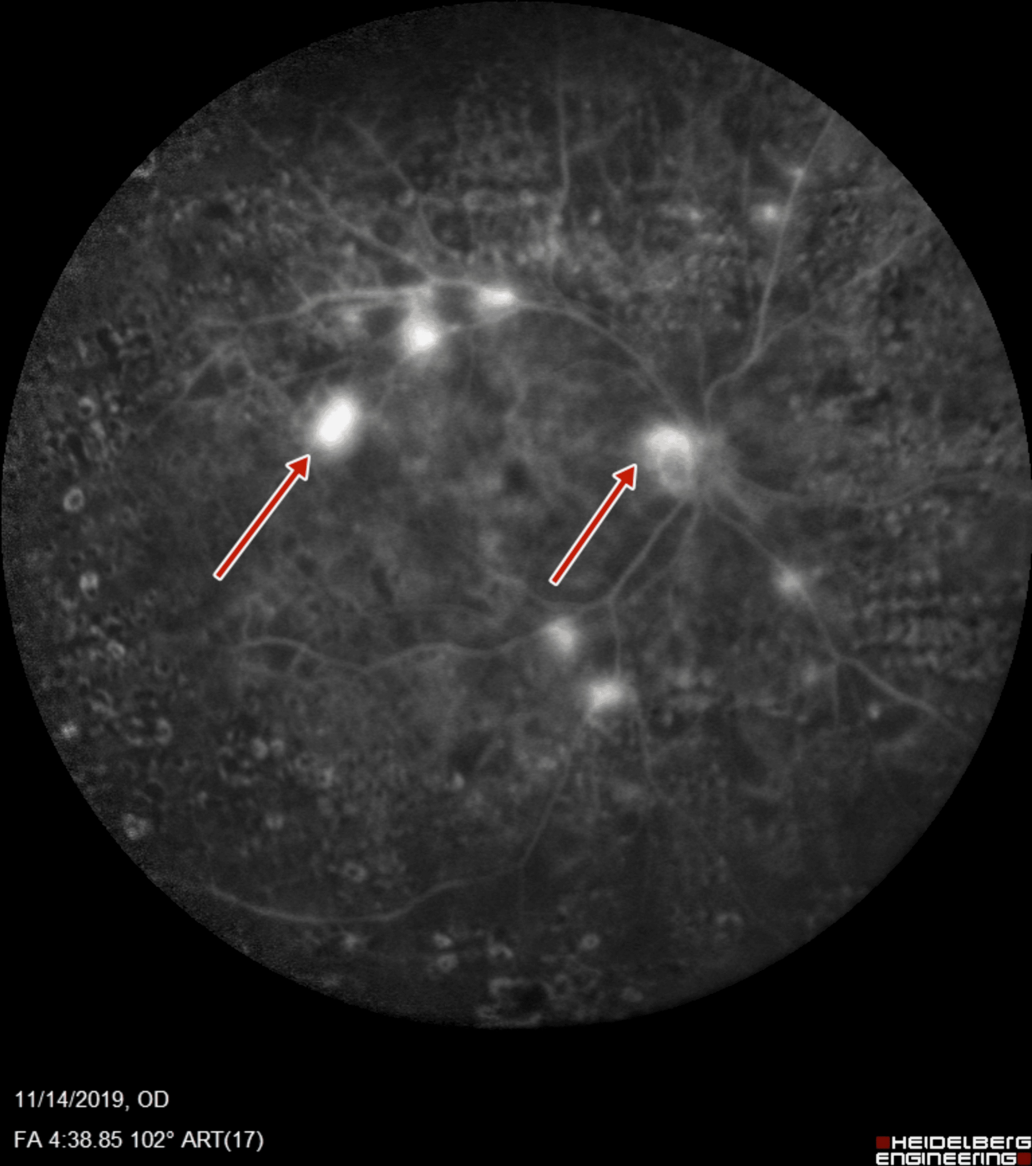
Right-eye wide field fluorescein angiography showing neovascularization of disc and retinal neovascularization along superotemporal arcade

**Figure 7:**
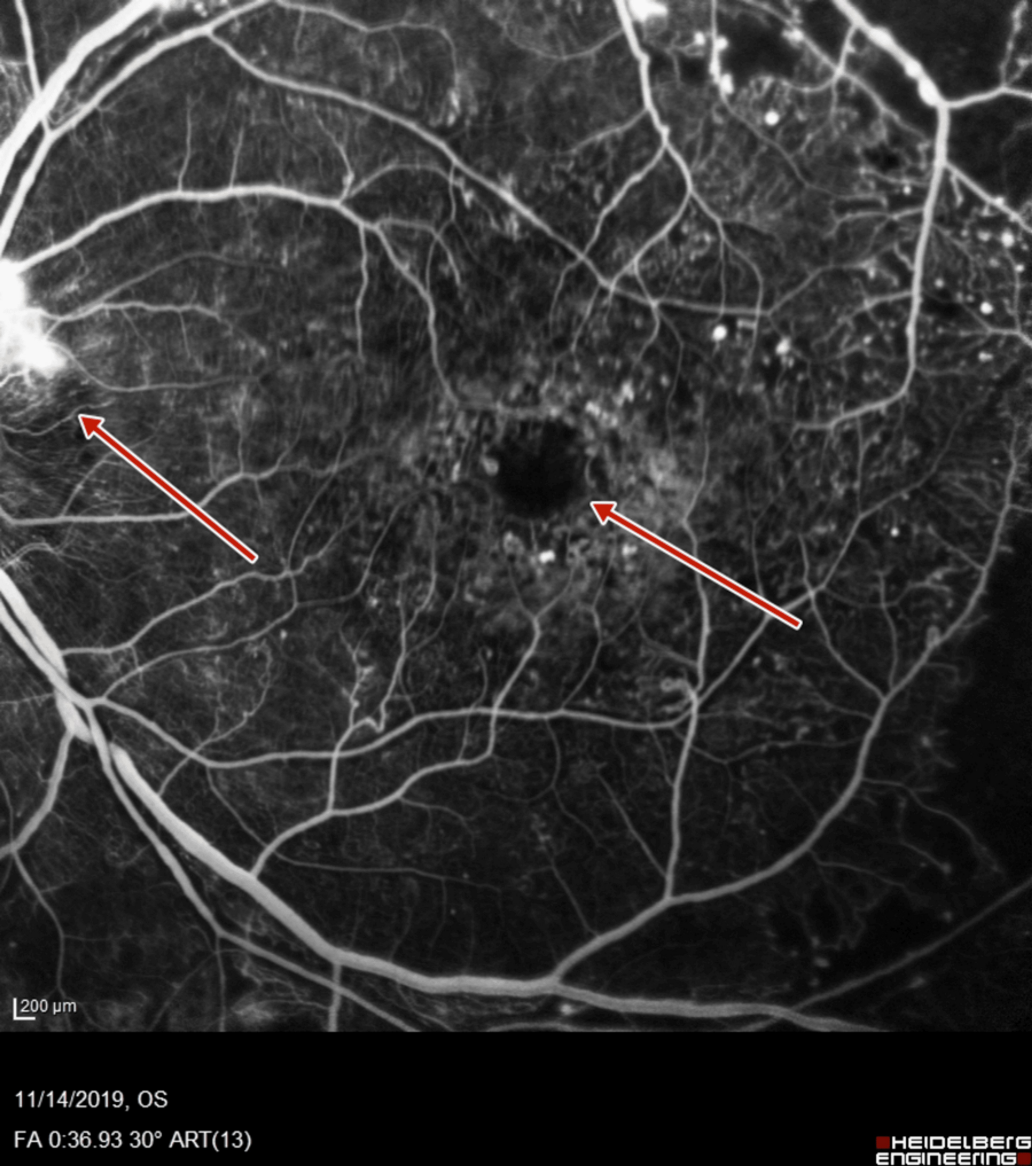
Left-eye fluorescein angiography showing neovascularization of disc and increased foveal avascular zone

**Figure 8:**
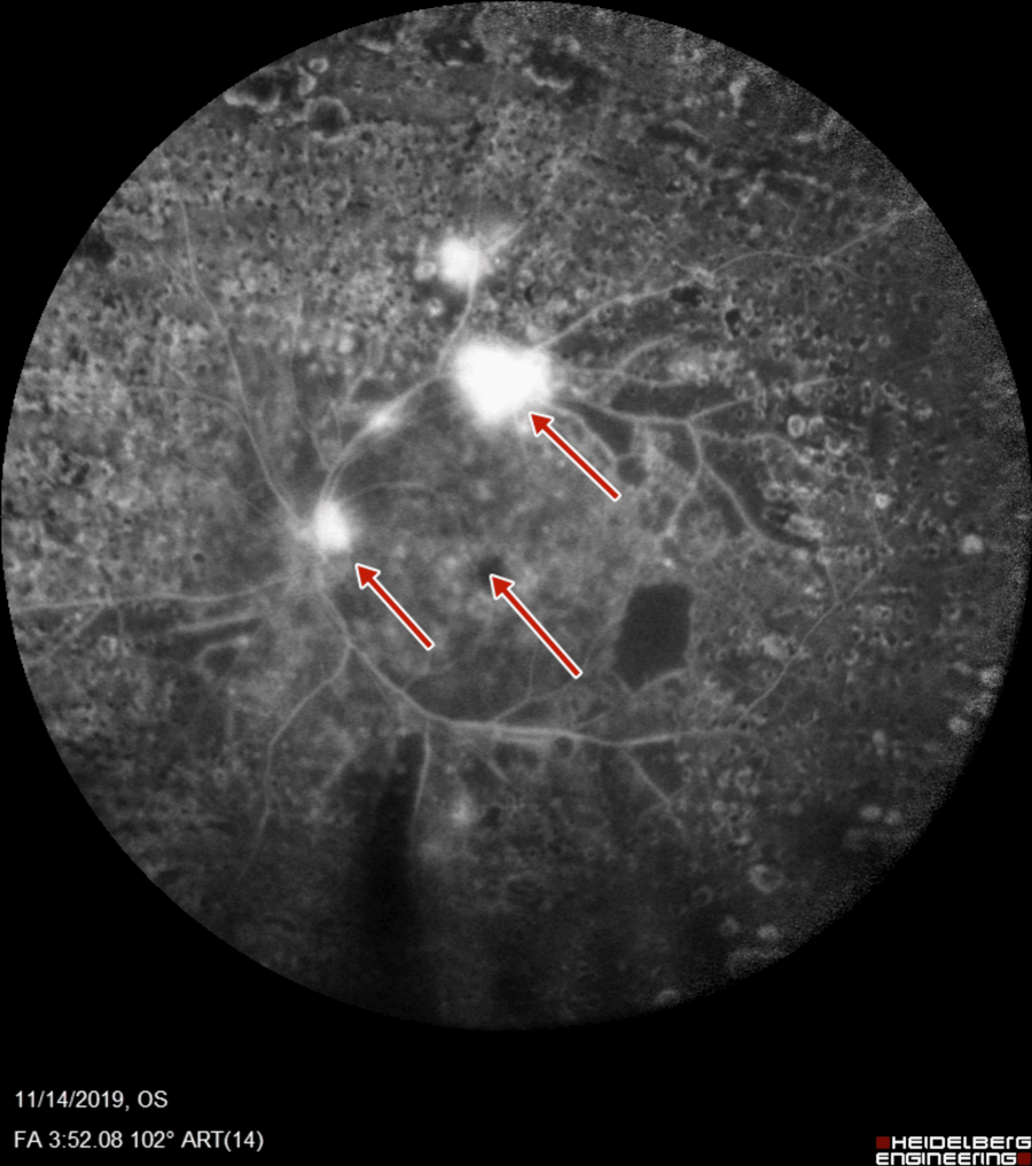
Left-eye fluorescein angiography showing neovascularization of disc, retinal neovascularization, and increased foveal avascular zone

Subsequent fluorescein angiography (FA) (Heidelberg retinal angiogram) was performed to verify the adequacy of PRP, which confirmed the presence of retinal neovascularization (NVE) and optic disc neovascularization (NVD), with features of PDR (Figures 5-8).

**Figure 9:**
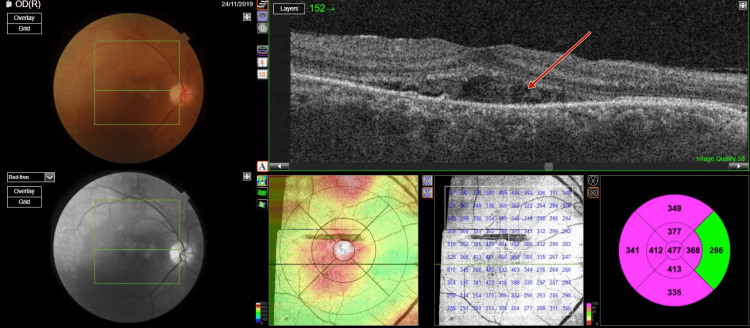
Right-eye post-panretinal photocoagulation optical coherence tomography showing exudative subretinal fluid at the fovea with hyperreflectivity suggestive of high protein content of the subretinal fluid

**Figure 10:**
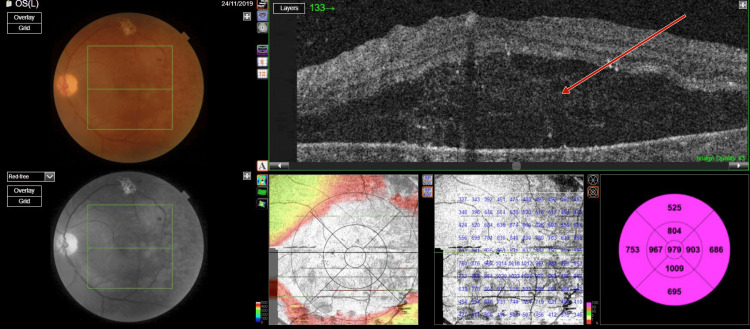
Left-eye post-panretinal photocoagulation optical coherence tomography showing exudative subretinal fluid at the fovea with hyperreflectivity suggestive of high protein content of the subretinal fluid

The patient underwent PRP in both eyes under the following laser parameters: 532 nm, 250-300 MW, 200 ms duration, and 200 μm spot size. There were 548 laser spots in the right eye and 576 laser spots in the left eye, with intensity ranging from grades 2 to 3. Post-PRP, the patient complained of visual loss, with a vision on a decimal scale of 0.4 in the right eye and 0.6 in the left eye. IOP was 12 mmHg in the right eye and 14 mmHg in the left eye, respectively. Both eye anterior segments were normal, and both eyes’ vitreous were quiet. The right eye fundus was suggestive of NVD at the disc, with lasered PDR and circumscribed elevation at the macula. In contrast, the left eye showed NVD and NVE, with laser marks on the retina and subretinal fluid at the macula, suggestive of exudative RD. OCT was performed, which confirmed the presence of subretinal fluid at the macula in both eyes, suggestive of exudative RD. The subretinal fluid at the macula in the right eye showed hyperreflectivity, consistent with the high protein content of the exudative fluid (Figures 9-10).

**Figure 11:**
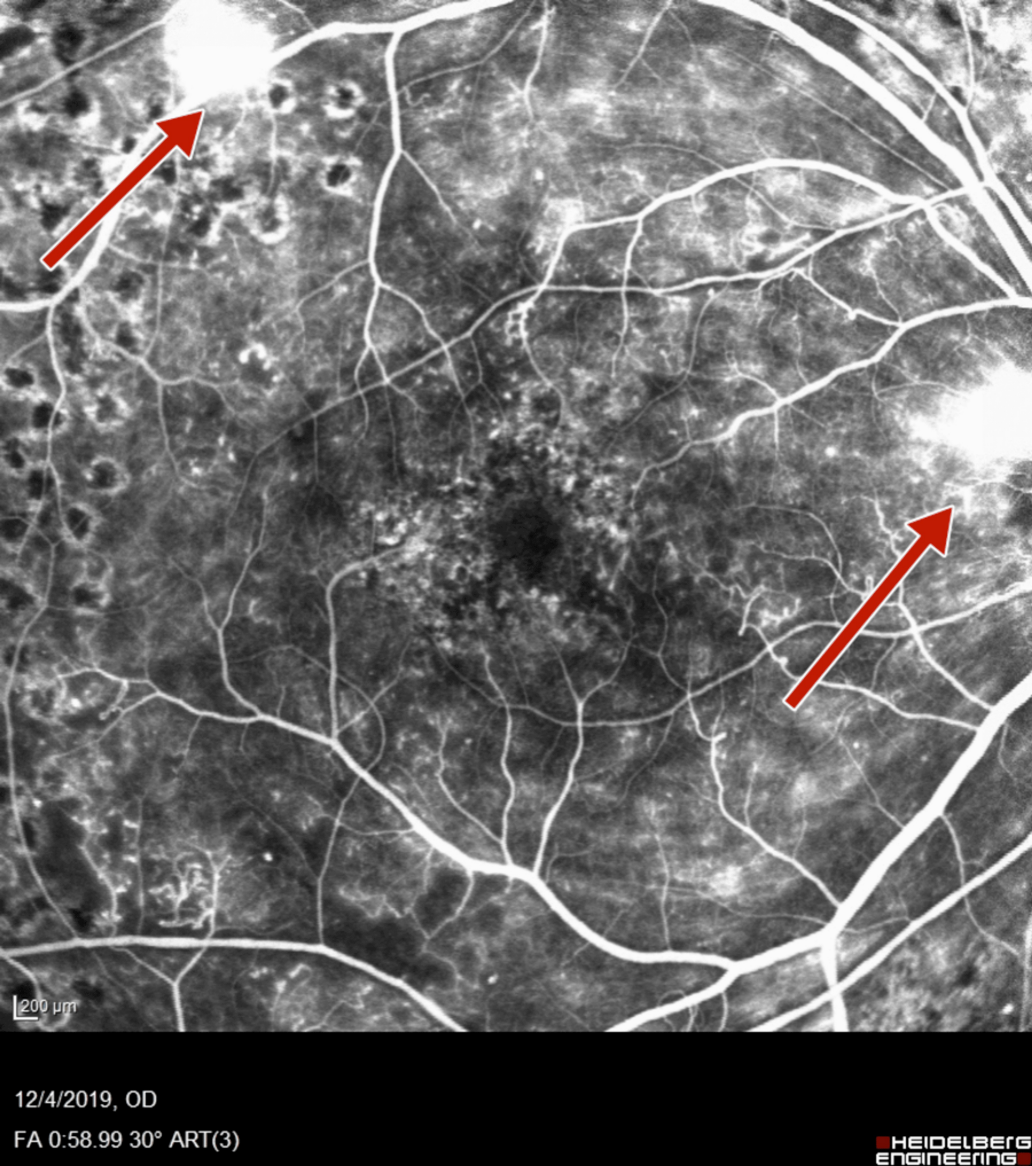
Right-eye post-panretinal photocoagulation fluorescein angiography suggestive of neovascularization of disc and retina

**Figure 12:**
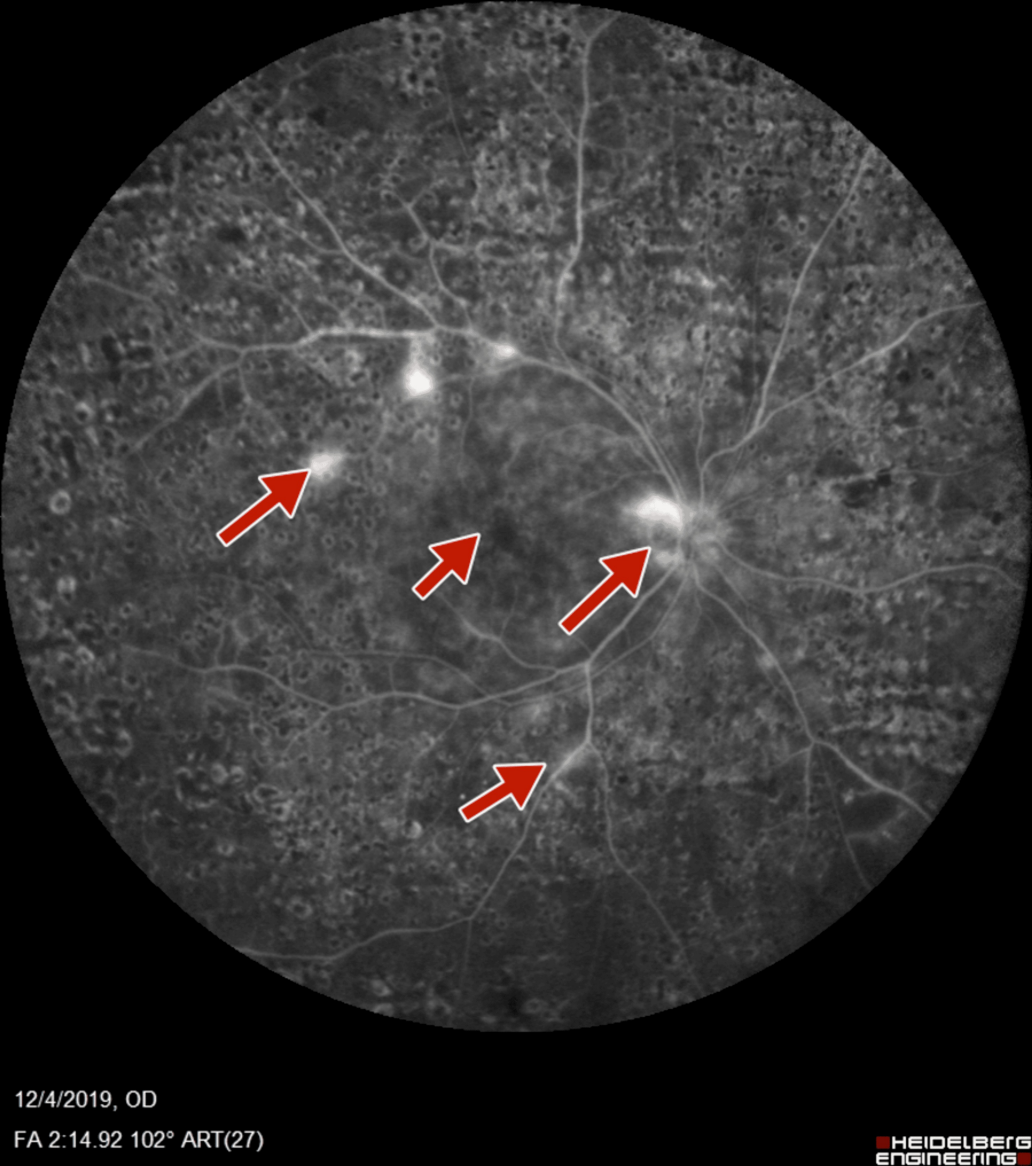
Right-eye post-panretinal photocoagulation wide-field fluorescein angiography suggestive of neovascularization of disc, as well as retina with staining at laser scars with increased foveal avascular zone

**Figure 13:**
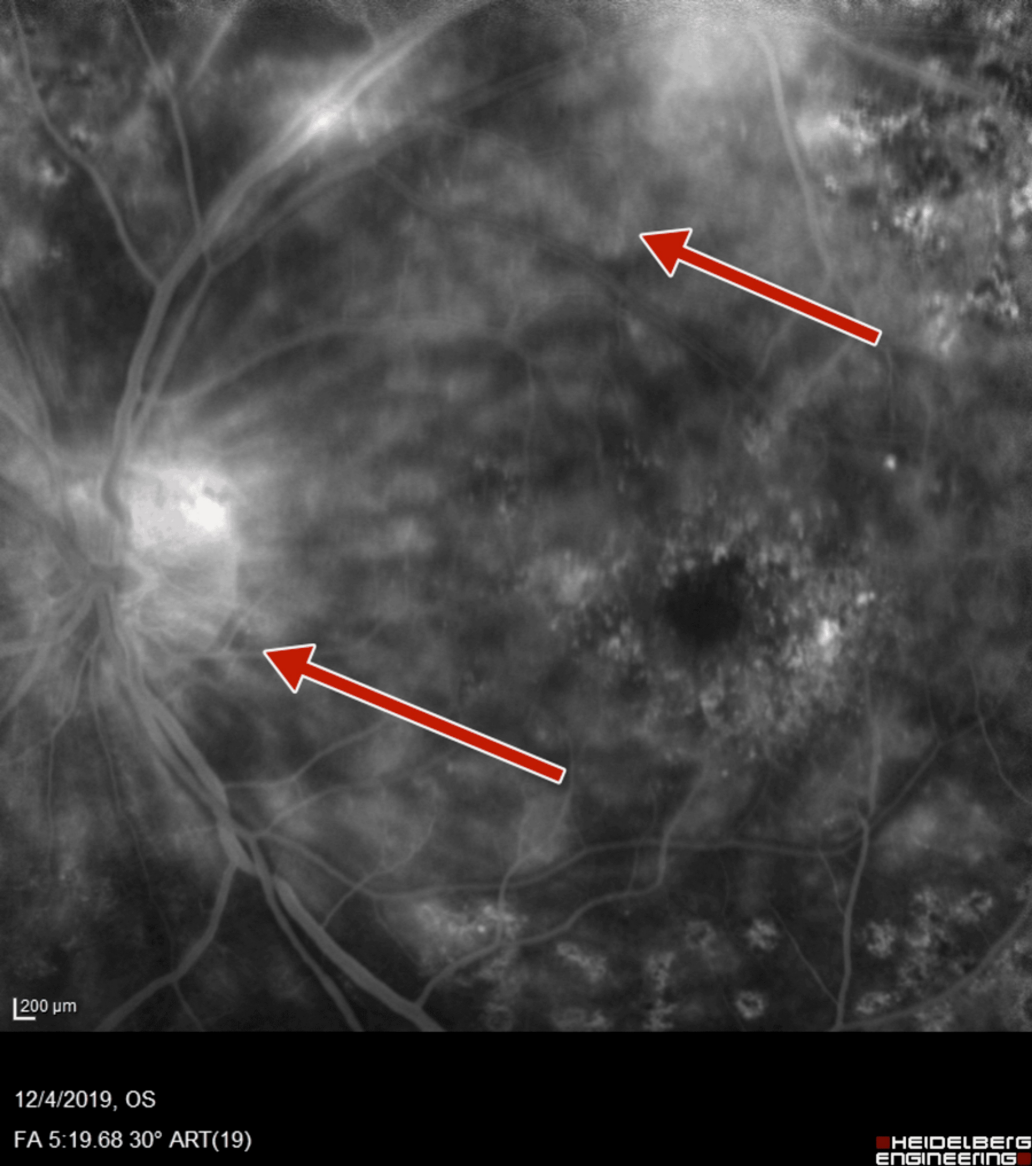
Left-eye post-panretinal photocoagulation fluorescein angiography suggestive of neovascularization of disc, with staining of the retinal blood vessels suggestive of loss of blood-retinal barrier

**Figure 14:**
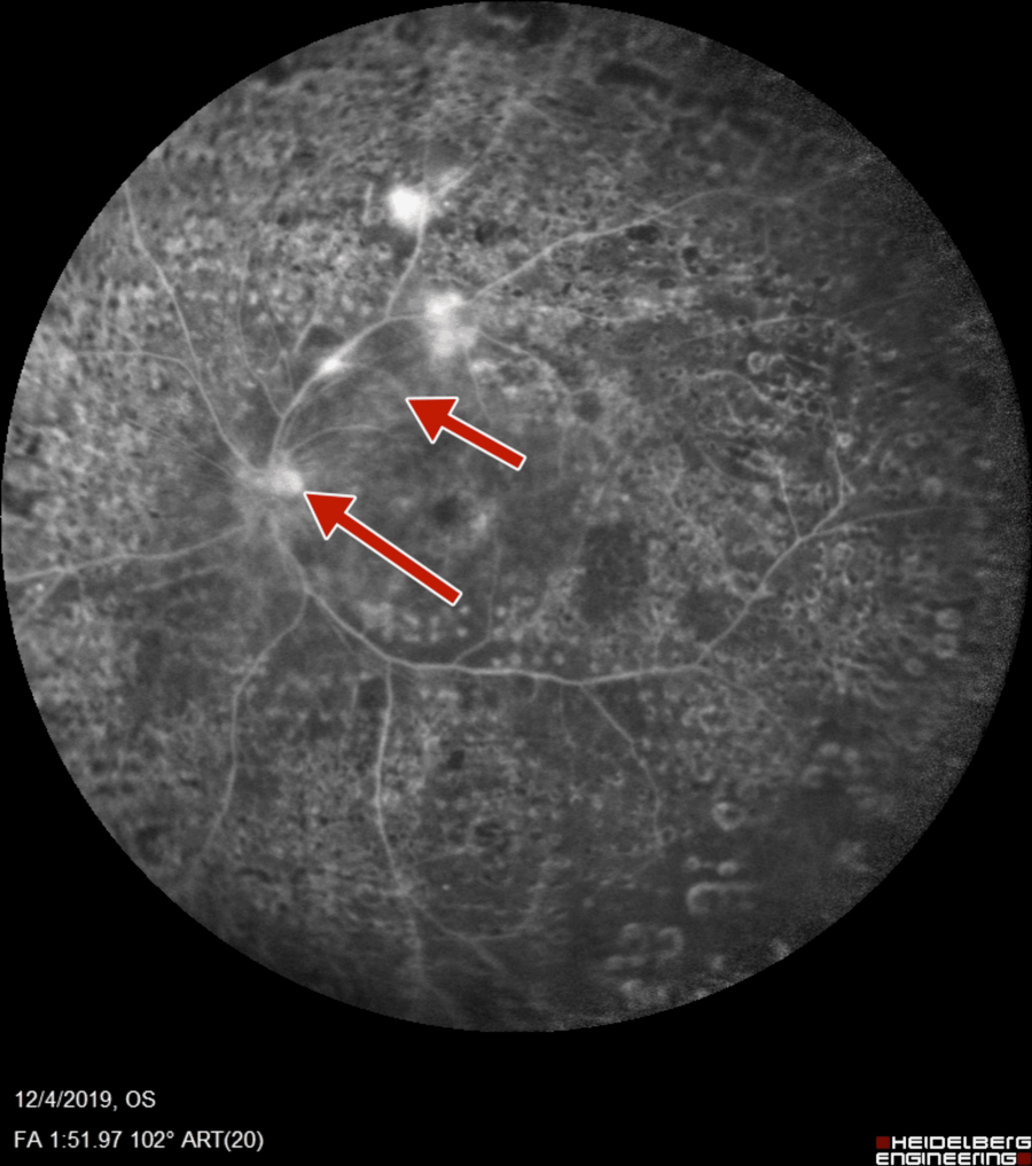
Left-eye post-panretinal photocoagulation wide-field fluorescein angiography suggestive of neovascularization of disc, with staining of the retinal blood vessels suggestive of loss of blood-retinal barrier

FA was suggestive of NVD, and diffuse staining of the vessels in the late phases suggested loss of the blood-retinal barrier (Figures 11-14).

**Figure 15:**
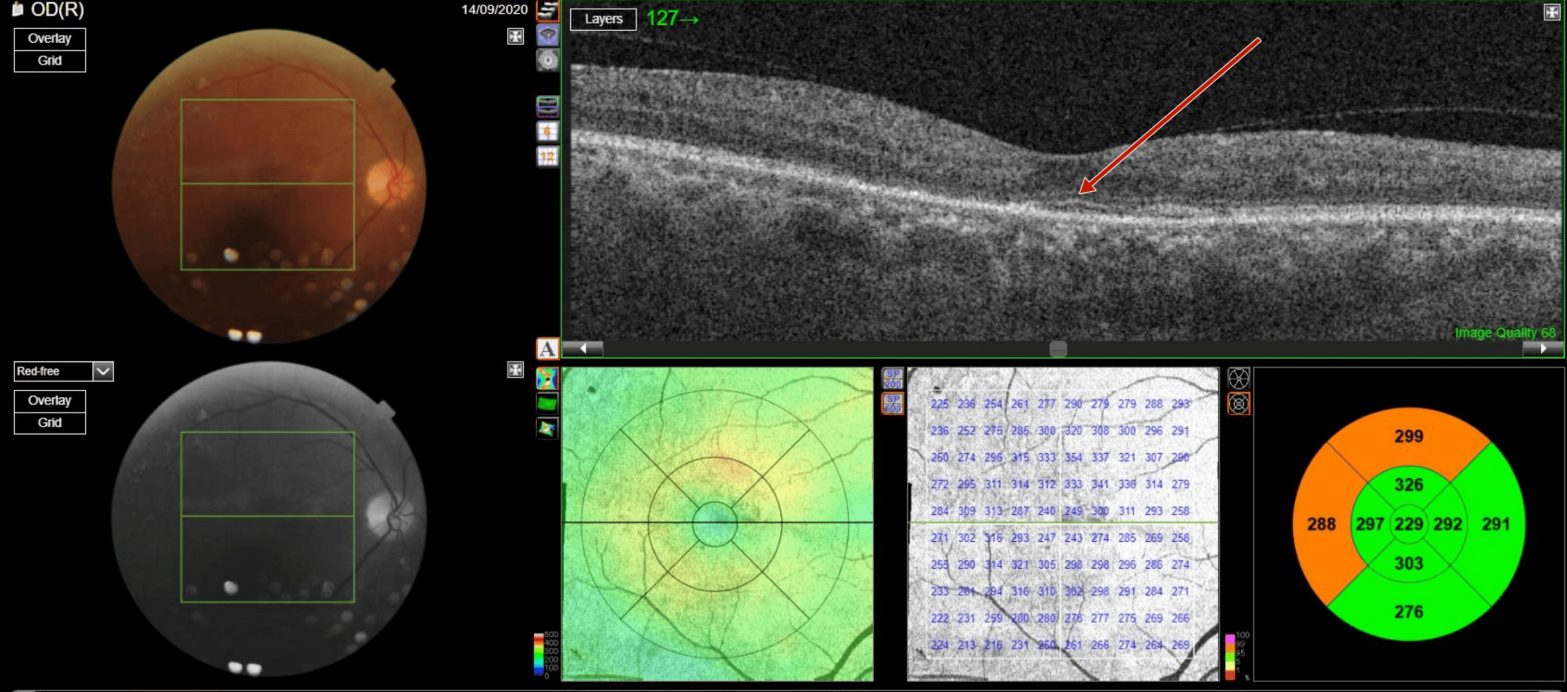
Follow-up right-eye optical coherence tomography scan showing complete resolution of subretinal fluid at macula

**Figure 16:**
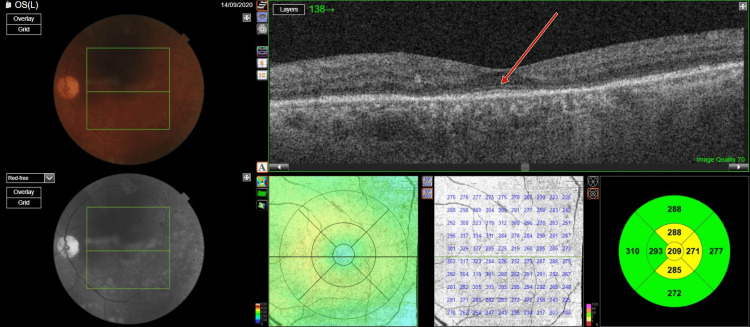
Follow-up left-eye optical coherence tomography scan showing complete resolution of subretinal fluid at macula

The patient was kept on nonsteroidal anti-inflammatory eyedrops. At a two-week follow-up appointment, his vision improved to 0.9 OU, the anterior segment was normal, and the fundus revealed lasered PDR with complete resolution of subretinal fluid in subsequent follow-up after one month (Figures 15-16).

Case 2

**Figure 17:**
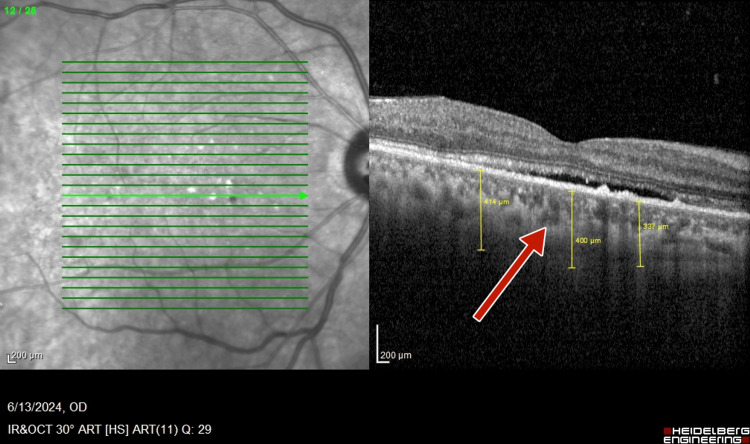
Right-eye optical coherence tomography showing trace subretinal fluid with serous pigment epithelial detachment with pachyhoroid

**Figure 18:**
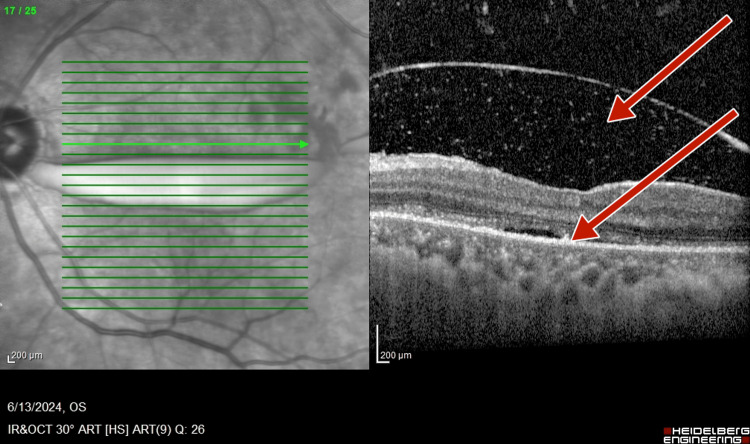
Left-eye optical coherence tomography showing trace subretinal fluid along with preretinal hemorrhage and incomplete posterior vitreous detachment

A 50-year-old diabetic male presented to us with reduced vision in his left eye for two days. He had renal dysfunction (blood creatinine = 2.5 mg/dL) and no history of retinal laser treatment, with an HbA1C of 9% (optimum level of HbA1C in diabetic patients: 7%), suggesting inadequate glycemic control. His presenting visual acuity on decimal charting was 0.8 in the right eye and 0.4 in the left eye, and his IOP was 12 mmHg in both eyes. Anterior segment examination revealed grade 2 nuclear sclerosis in both eyes, with the rest of the findings being normal. Fundus examination revealed features suggestive of PDR in both eyes (OU). The patient underwent an OCT (Heidelberg, Kennesaw, GA, USA) examination, which revealed subfoveal serous PED and trace subretinal fluid in both eyes. His right eye had a subfoveal choroidal thickness of 400 µm, suggestive of pachychoroid (Figures 17-18).

**Figure 19:**
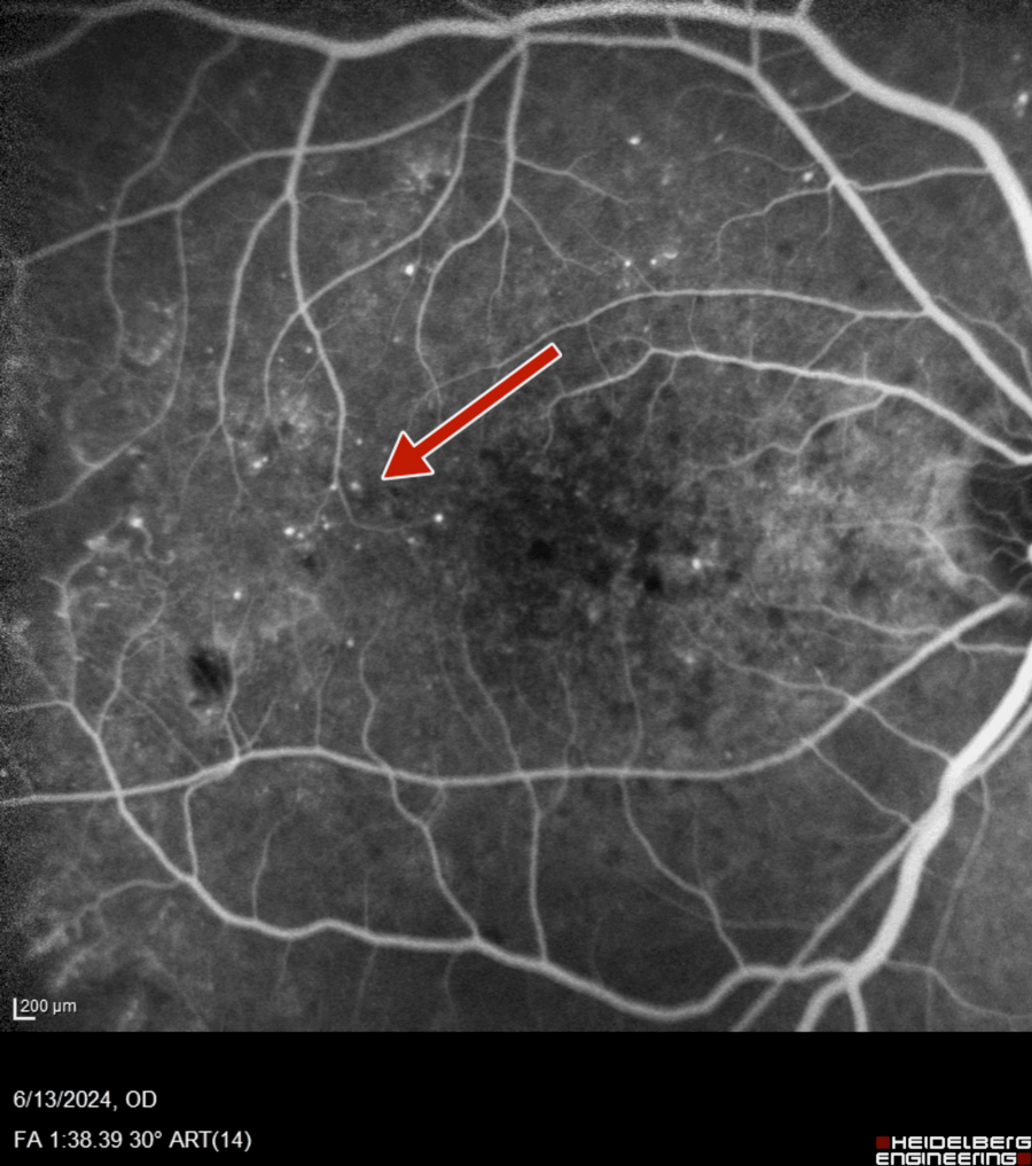
Right-eye fluorescein angiography showing microaneurysms

**Figure 20:**
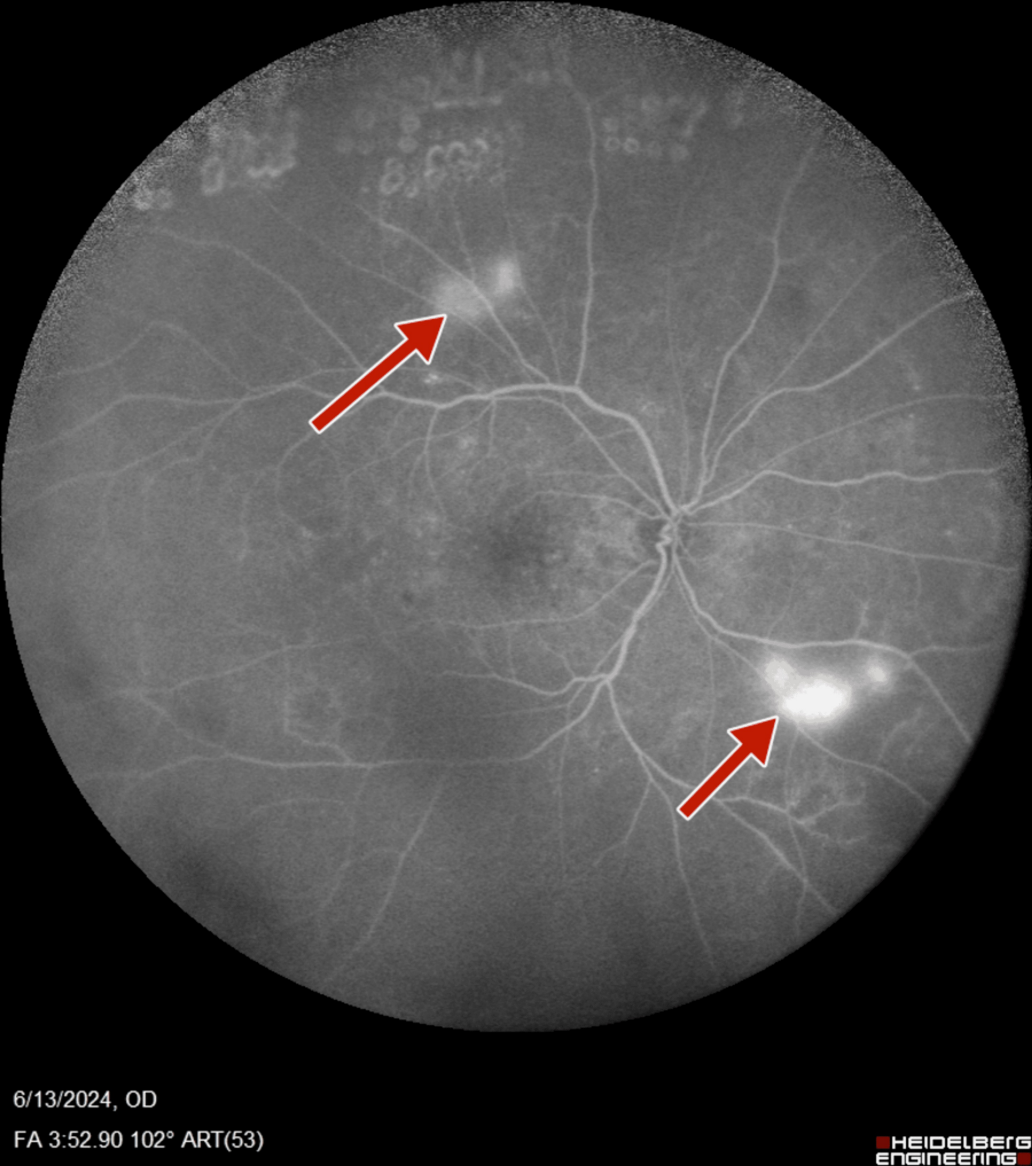
Right-eye late-phase fluorescein angiography showing retinal neovascularization

**Figure 21:**
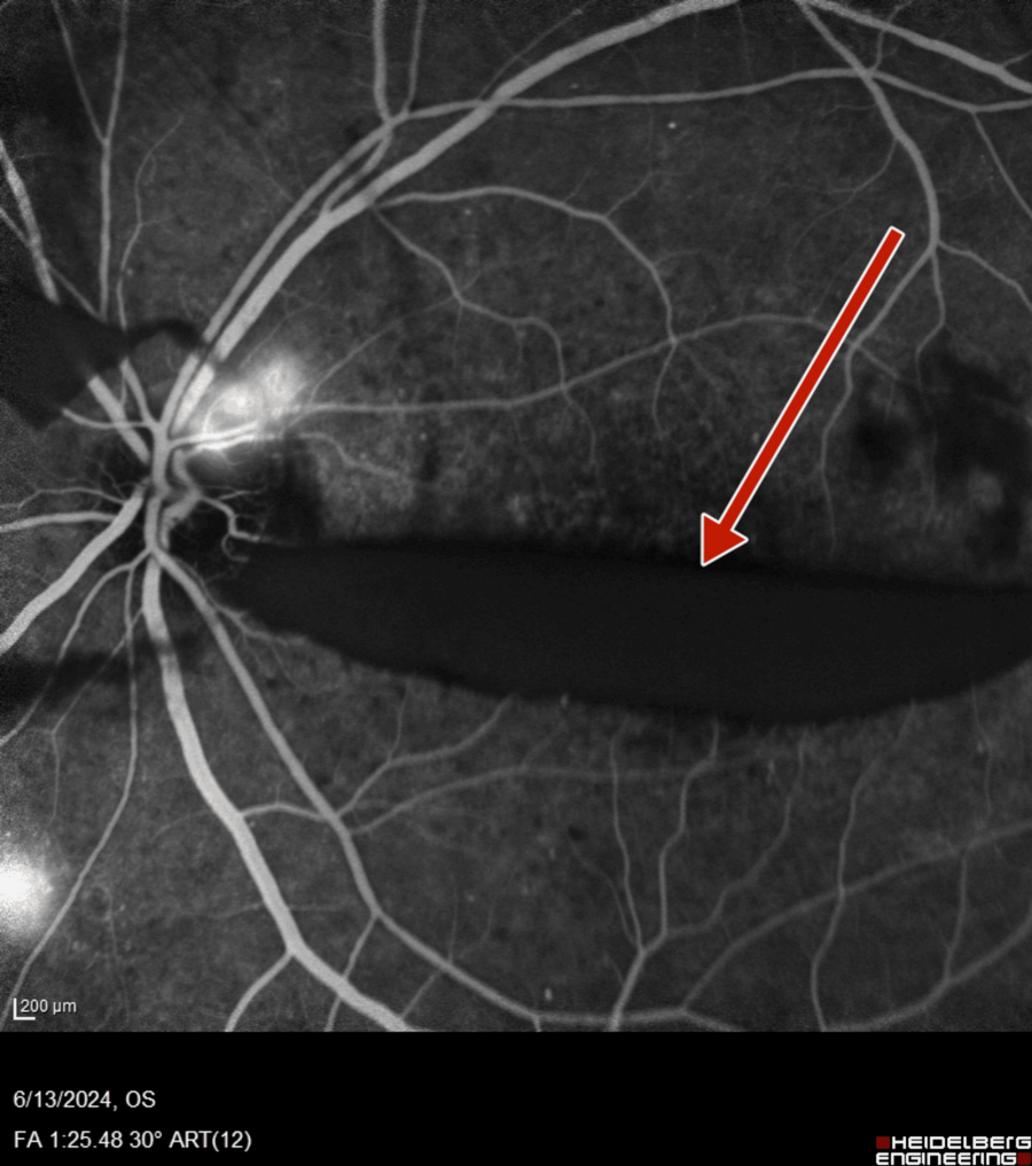
Left-eye fluorescein angiogram showing boat-shaped premacular hemorrhage

**Figure 22:**
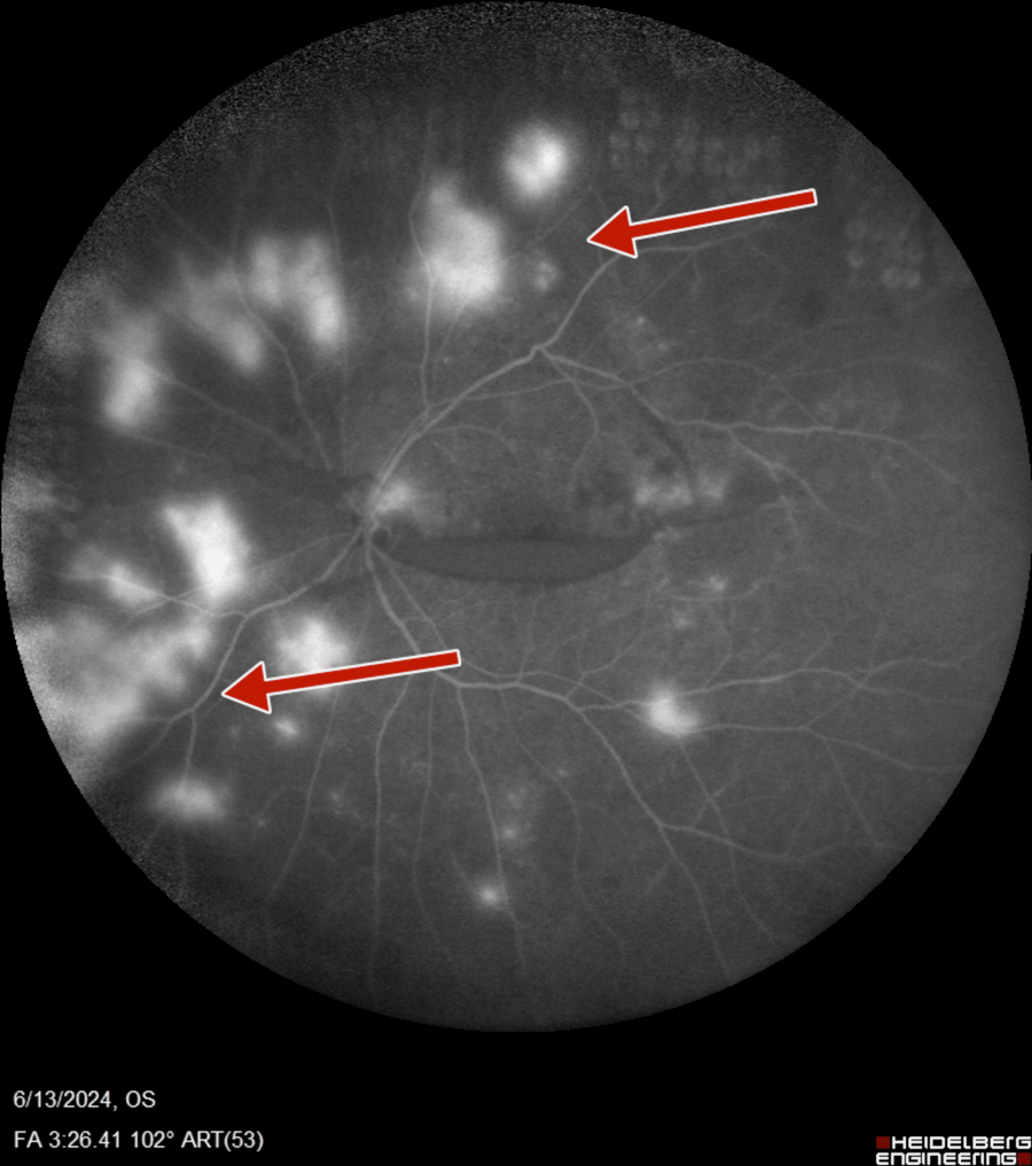
Left-eye late-phase fluorescein angiogram showing multiple retinal neovascularization

Subsequent FA (Heidelberg retinal angiogram) confirmed the presence of NVE in both eyes and blocked fluorescence corresponding to boat-shaped preretinal hemorrhage in the left eye (Figures 19-22).

**Figure 23:**
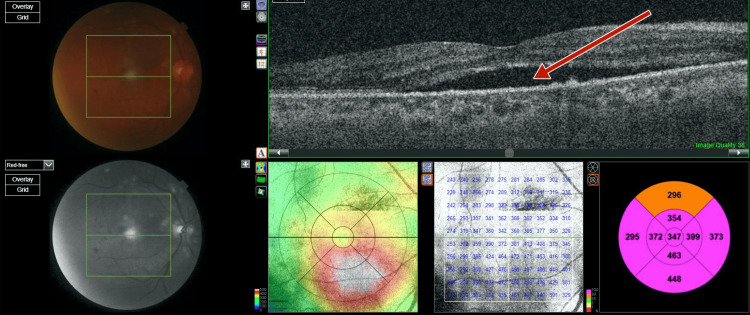
Right-eye optical coherence tomography showing subretinal fluid at macula due to exudative retinal detachment

**Figure 24:**
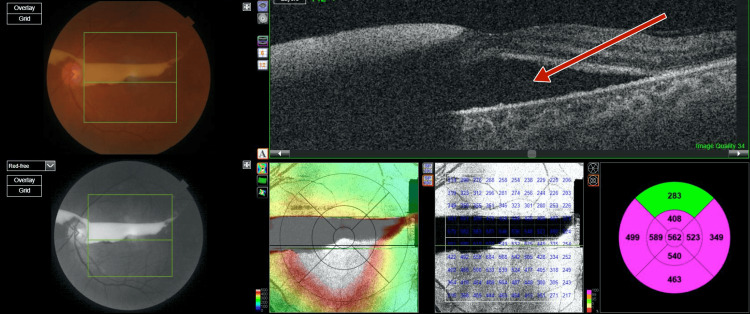
Left-eye optical coherence tomography showing subretinal fluid at macula due to exudative retinal detachment

The patient underwent PASCAL laser photocoagulation in both eyes under the following laser parameters: 532 nm, 250-300 MW, 200 ms duration, and 200 µm spot size. There were 588 laser spots in the right eye and 590 laser spots in the left eye, with intensity ranging from grades 2 to 3. Post-PRP, the patient complained of visual loss, with visual acuity on the decimal scale of 0.6 in the right eye and 0.2 in the left eye, whereas IOP was 12 mmHg in both eyes. Both eye anterior segments were normal, and both eyes' vitreous were quiet. Both eyes showed features suggestive of NVE and laser marks, along with circumscribed elevation at the macula. OCT was performed, which confirmed the presence of subretinal fluid at the macula in both eyes, suggestive of exudative RD (Figures 23-24).

The patient was kept on nonsteroidal anti-inflammatory eyedrops and was reassured. At a two-week follow-up appointment, his vision improved to 0.9 OD and 0.5 OS; the anterior segment was normal, and the fundus revealed lasered PDR with a resolution of exudative RD by three weeks. Table 1 illustrates the comparison of both the patients.

**Table 1:** Comparison of patient 1 and patient 2 PRP: Panretinal photocoagulation

Parameters	Patient 1		Patient 2	
	Right eye	Left eye	Right eye	Left eye
Pre-PRP vision (on decimal scale)	0.9	0.9	0.8	0.4
Post-PRP vision (on decimal scale)	0.4	0.6	0.6	0.2
Choroidal thickness (µm)	415	461	400	Pachychoroid
Visual acuity at 2 weeks	0.9	0.9	0.9	0.5

## Discussion

Diabetic choroidopathy and pachychoroid

Changes in diabetic choroidopathy often precede retinopathy, with the identification of its characteristics having pathogenetic and prognostic implications for the progression of retinopathy [5]. There are reports of increased choroidal thickness (pachychoroid) or reduced choroidal thickness (leptochoroid) associated with diabetic retinopathy [6-8].

The pachychoroid spectrum is associated with many retinal pathologies, such as central serous chorioretinopathy, idiopathic choroidal vasculopathy, Vogt-Koyanagi-Harada syndrome, and serous PED [9]. Indocyanine green angiography-based studies have shown increased choroidal hyperpermeability associated with these clinical entities [10]. There have also been reports of diabetic macular edema with submacular detachment associated with increased choroidal thickness [11]. Pachychoroid pigment epitheliopathy and CSR are also reported in cases of frequent injections of intravitreal steroids [12], whereas reduction in choroidal thickness has been reported in young patients with PDR and renal dysfunction [13], as well as in those receiving anti-vascular endothelial growth factor (anti-VEGF) injections [14].

Choroidal thickness decreases with the progression of diabetic retinopathy due to progressive choroidal hypoperfusion. Choroidal hypoperfusion occurs with the accumulation of periodic acid-Schiff stain-positive material within the lumen of the choriocapillaris [15,16]. Studies have shown that the choroidal vascularity index is reduced in diabetic choroidopathy and with the progression of diabetic retinopathy [8]. In addition, the choroidal vascularity index decreases with PDR and after PRP, which is associated with a reduction in choroidal thickness [17].

PRP and exudative RD

PRP is the mainstay of treatment for PDR, in which PASCAL is a popular laser mode due to its multispot delivery, reduced laser time, decreased collateral damage, and reduced post-laser visual field defects [18]. However, PRP is associated with certain side effects, such as cataracts, macular edema, and RD [3]. Exudative RD post-laser is known to develop in young patients with poor glycemic control [3,19], but it can occur independent of gender, age, glycemic control, vitreous status, and with a moderate number of spots [4]. Exudative RD is also reported in nanophthalmos [20], as well as in patients with pachychoroid and hypotony maculopathy post-trabeculectomy for diabetic neovascular glaucoma [21].

Although the mechanism of formation of exudative RD in these cases and our case report might be the same, in our case series, it was the laser-induced inflammation that acted as a trigger factor.

In our case series, it was evident that both of our patients had lasered unstable PDR with serous PED at the macula in both eyes, along with pachychoroid in both cases. Serous PED is known to be associated with pachychoroid [9]. In our first case, the patient exhibited normal renal function, and in the second case, renal dysfunction was present, with no other comorbidities except for thickened choroid. In our first case, the exudative RD may have been due to laser-induced retinal pigment epithelial dysfunction, whereas this factor was not present in our second case. PRP causes an increase in choriocapillary density in the macular area and an increase in choroidal blood flow due to redistribution of blood supply from the occluded capillary plexus at one and six months [22]. In contrast, in our case series, exudative RD was seen a few days after PRP. We hypothesize that in both cases, laser-induced inflammation led to a further increase in choroidal thickness, worsening the choroidal hyperpermeability, which overwhelmed the retinal pigment epithelial function, leading to exudative RD. Although there have been reports of post-PRP exudative RD, what makes our case report unique is the identification of pachychoroid as one of its risk factors.

We also suggest using anti-VEGF agents in conjunction with laser PRP in such patients. However, it should be kept in mind that anti-VEGF injections should be administered after ruling out vitreoretinal traction, as they can hasten the development of tractional RD in patients with PDR [23]. We also suggest using anti-VEGF agents in conjunction with laser PRP in such cases. Anti-VEGF agents, such as aflibercept, are known to reduce choroidal thickness [24] and can prove useful in preventing this complication if used in conjunction with or before laser PRP.

There have been reports of choroidal and retinal exudative changes after extensive endolaser PRP mimicking central serous retinopathy; however, there is no reported discussion on pachychoroid as a predictive factor for post-laser exudative RD in the literature [25].

## Conclusions

Choroidal thickness can vary in diabetic retinopathy. Identification of pachychoroid before laser PRP is unique to this case report. As panretinal laser photocoagulation, in the presence of a pachychoroid, can be a risk factor for exudative RD, identification of the pachychoroid prior to laser treatment can increase the predictability of exudative RD. We also consider encouraging our colleagues to report post-laser exudative RD in pachychoroid cases, which can pave the way to a randomized controlled trial to validate our hypothesis. The option of using anti-VEGF agents in the prevention of exudative RD in such cases would also require further study.
